# Synthesis and Evaluation of Novel Biased μ-Opioid-Receptor (μOR) Agonists

**DOI:** 10.3390/molecules24020259

**Published:** 2019-01-11

**Authors:** Mengjun Ma, Jialin Sun, Menghua Li, Zixing Yu, Jingchao Cheng, Bohua Zhong, Weiguo Shi

**Affiliations:** 1 State Key Laboratory of Toxicology and Medical Countermeasures, Beijing Institute of Pharmacology & Toxicology, 27 Tai-Ping Road, Beijing 100850, China; mengjunma@126.com (M.M.); monkeysjl@163.com (J.S.); yuyuzx1990@163.com (Z.Y.); chengjingchao3017@126.com (J.C.); 2 National Glycoengineering Research Center and Shandong Provincial Key Laboratory of Carbohydrate Chemistry and Glycobiology, Shandong University, 27 Shanda Nan Lu, Jinan 250100, China; lmh0920@126.com; 3 TaizhouHuayuan Med Tech Company LTD, 1 Yaocheng Avenue, Taizhou 225312, China

**Keywords:** μOR-biased agonist, PZM21, Gi signaling pathway, β-arrestin-2 recruitment, structure–activity relationship

## Abstract

‘Biased’ ligands of G protein-coupled receptors (GPCRs) represent a type of promising analgesic with reduced on-target side effects. PZM21, a potent μ-opioid-receptor (μOR)-biased agonist with a new chemical scaffold compared to classic opioids, has been identified as a therapeutic lead molecule for treating pain. In the current study, novel PZM21 analogues were synthesized and evaluated for their in vitro and in vivo efficacy. Novel compound **7a** and PZM21 demonstrated undetectable β-arrestin-2 recruitment, however, their analgesic effects need to be further confirmed. Compounds **7b**, **7d**, and **7g** were stronger analgesics than PZM21 in both the mouse formalin injection assay and the writhing test. Compound **7d** was the most potent analogue, requiring a dose that was 1/16th to 1/4th of that of PZM21 for its analgesic activity in the two assays, respectively. Therefore, compound **7d** could serve as a lead to develop new biased μOR agonists for treating pain.

## 1. Introduction

The discovery of safer and more effective analgesics without the drawbacks of classic opioids is urgently needed. ‘Biased’ ligands of G protein-coupled receptors (GPCRs) represent a type of promising molecule with a specific ability to cherry pick the beneficial rather than the deleterious signaling pathways activated by the μ-opioid-receptor (μOR) so that on-target toxicity is reduced [1,2,3]. Recent studies have suggested that some GPCR ligands exhibit an “unbalanced effect” when activating signaling pathways. They can bind to specific receptor forms [4,5] or selectively bind to different types of G protein subunits or even β-arrestin, thereby biasing the cytoplasmic signal to a certain pathway. These ligands are named ‘biased agonists’ or ‘biased ligands’ [6,7,8]. Previous research has shown that opioid-induced analgesia results from μOR signaling through the G protein G_i_; while many side effects, including respiratory depression and constipation, may be transmitted through β-arrestin pathway signaling, which occurs downstream of μOR activation [9,10,11].

**Figure 1:**
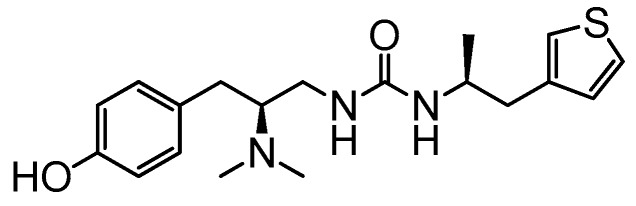
The chemical structure of PZM21.

PZM21 (Figure 1) is a potent biased μOR agonist with a structurally distinct chemical scaffold unrelated to known opioids. It was initially identified from over three million molecules by computational docking against the μOR structure; therefore, it is a promising therapeutic lead for pain relief [12,13]. Although the potency and biased action of PZM21 have been partially confirmed [13,14], the detailed structure–activity relationship of this type of compound needs to be further investigated.

**Figure 2:**
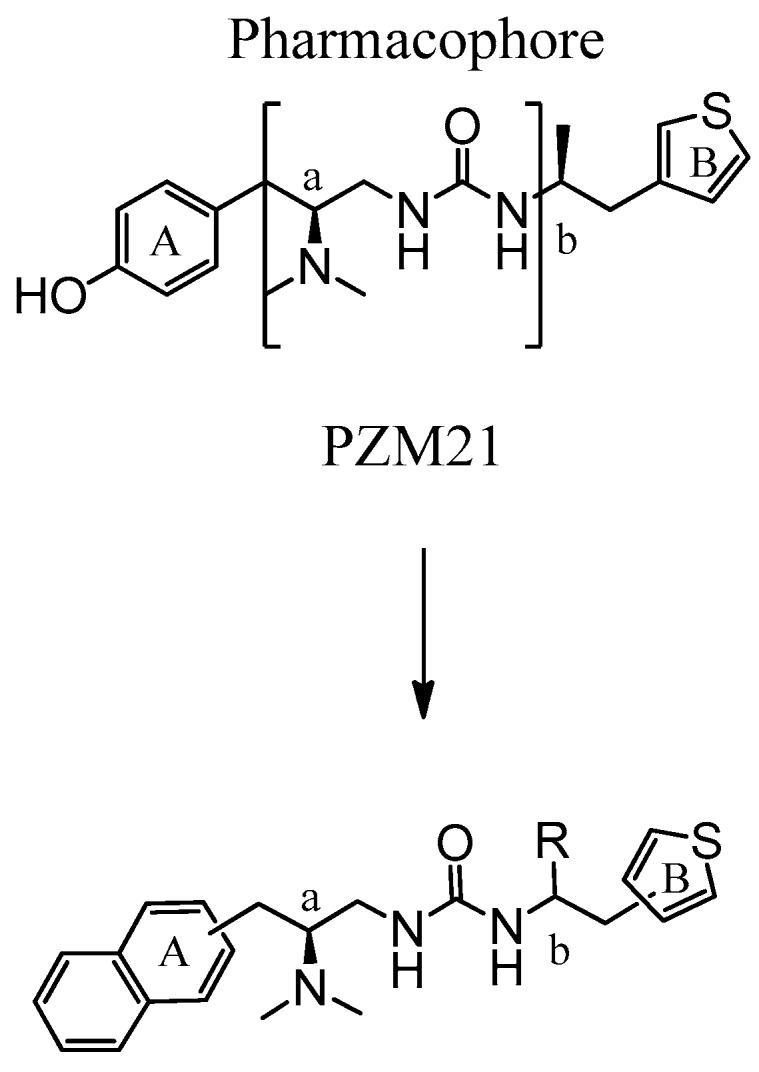
The design of novel PZM21 analogues.

Here, synthetic structure optimization of PZM21 was performed by introducing an aromatic naphthalene scaffold to replace the benzene ring of PZM21, leaving the specific dimethyl amino and urea pharmacophore groups unchanged. Additionally, the position of the thiophene ring substituent and the chirality of the methyl group were investigated. A series of PZM21 analogues (Figure 2) were synthesized and assessed for their in vitro and in vivo activities.

## 2. Results and Discussion

### 2.1. Synthesis

**Scheme 1:**
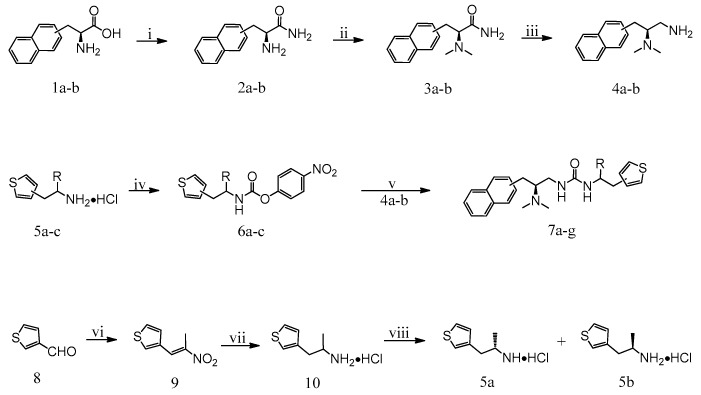
Synthesis of compounds **7a**–**g**. Reagents and Conditions: (i) SOCl_2_, CH_3_OH, 0 °C to rt, 12 h, NH_4_OH, 12 h; (ii) CH_2_O, H_2_/Pd-C, CH_3_OH, rt, 0.4 MPa, 5 h; (iii) BH_3_, THF, reflux, 15 h; (iv) 4-nitrophenyl chloroformate, triethylamine, THF, 0 °C to rt, 6 h; (v) triethylamine, CH_3_CN, 60 °C to −80 °C, 2 h; (vi) nitroethane, HCOOH/ethanolamine, 90 °C; (vii) LiAlH_4_, THF, reflux, 30 min; (viii) di-*p*-anisoyl-D-tartaric acid, di-*p*-anisoyl-l-tartaric acid, acetonitrile, rt.

As shown in Scheme 1, target compounds **7a**–**g** were synthesized in five steps. First, the amino acids were converted to amino amides, which were then subjected to reductive amination with formaldehyde. Next, the carbonyl group was reduced by borane to obtain intermediates **4a** and **4b**. The raw materials **5a**–**d** were, respectively, reacted with 4-nitrophenyl chloroformate to obtain **6a**–**d**, which were reacted with **4a** or **4b** to obtain final products **7a**–**g**.

### 2.2. The Selective G_i_-Biased μOR Agonist Activities and the β-Arrestin Recruitment Assay In Vitro

#### 2.2.1. μOR G_i/o_-Mediated cAMP Inhibition

The μOR agonist activities of **7a**–**g** were tested in a G_i/o_ signaling assay. To measure the activation of μOR by the G_i/o_-mediated signaling pathway, we detected the changes of intracellular cAMP content as the activity of adenosine cyclase can be inhibited by the combination of μOR with its agonist, which leads to a decrease in the intracellular cAMP concentration. The G_i/o_ signaling assay showed potent μOR agonist activity for new compounds **7b**, **7d**, **7e**, and **7g**, as well as PZM21 (Table 1). The potencies of **7b** (EC_50_ = 91.14) and **7e** (EC_50_ = 82.43) are similar to that of PZM21 (EC_50_ = 52.41), while **7d** and **7g** are weaker than PZM21.

#### 2.2.2. β-Arrestin Recruitment Assay

To determine whether these novel compounds activate μOR, we tested them in β-arrestin-2 recruitment assays. The results are shown in Table 2. PZM21 and **7a** showed undetectable β-arrestin-2 recruitment in the NanoBit assay. Unfortunately, **7b**, **7d**, **7e**, and **7g** showed potent β-arrestin recruitment.

### 2.3. Analgesic Activities of the Novel Compounds in a Formalin Injection Nociception Assay

Analgesia to a formalin injection was carried out as described previously [13]. The tested compounds and PZM21, at dosages of 20 mg/kg and 40 mg/kg, were injected subcutaneously, respectively. As shown in Table 3, all tested compounds produced sustained analgesia in phase 2 of the formalin injection assay (*p* < 0.001; compared with the vehicle group). The percentages of analgesia of **7b**, **7d**, **7e**, and **7g** were higher than that of PZM21 at both dosages. In particular, **7d** showed the most potent analgesic effect of 100% at both dosages, demonstrating that a lower dosage should be tested.

**Figure 3:**
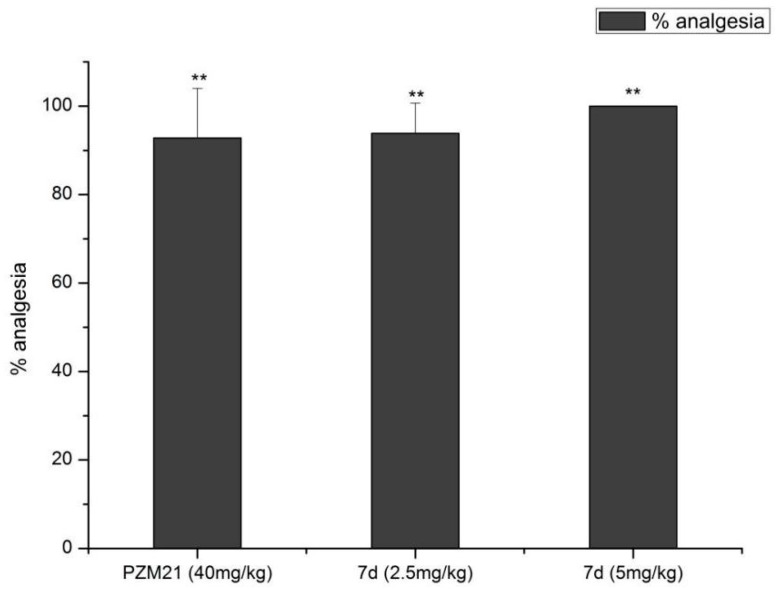
Activities of compound **7d** in the formalin injection assay. Values are the mean ± standard deviation (*n* = 8). **p < 0.001 versus control.

Further investigation revealed that compound **7d** exhibited 100% and 93.9% analgesia upon subcutaneous injection at 5 mg/kg and 2.5 mg/kg, respectively (Figure 3). These effects were more potent than that of PZM21, which showed 92.8% analgesia upon subcutaneous injection at 40 mg/kg.

### 2.4. Analgesic Activities of the Novel Compounds in the Writhing Test

The analgesic results of the novel compounds in the writhing test are shown in Table 4. Consistent with their activities in the formalin injection assay, **7b**, **7d**, and **7g** displayed stronger analgesia in the mouse writhing assay than PZM21. Additionally, compound **7a** showed a higher percentage of analgesia than PZM21. The low dose of **7d** (2.5 mg/kg) yielded a higher (92.7%) analgesic response than 10 mg/kg PZM21 (45.7%).

### 2.5. Discussion

The μOR-biased agonist PZM21 represents an attractive new chemical scaffold, which is unrelated to known opioids, with reduced on-target toxicity. Therefore, its structure-activity relationship needs to be investigated in detail. The tertiary amine and urea groups are unique to these novel compounds and are not present in other opioid agonists like morphine and fentanyl. Thus, we kept these core groups unchanged and introduced a naphthalene group to replace the phenolic hydroxyl of PZM21 in order to exploit the effects of steric bulk and hydrophobicity. Moreover, the requirement of specific stereoisomers for efficacy and the chemical position of the thiophene ring were also studied.

First, we tested the in vitro activities of the designed compounds and PZM21. Similar to PZM21, the novel compounds strongly activated G_i/o_. Unfortunately, most of the novel compounds also showed high levels of β-arrestin recruitment; only **7a** and PZM21 showed undetectable β-arrestin-2 recruitment in the assay. Next, we measured the analgesic responses of the novel compounds using different animal models for pain. Intriguingly, we observed no analgesic effect for PZM21 or the novel compounds in a mouse hotplate assay, even at a high dose of 100 mg/kg. These findings were inconsistent with those reported for PZM21 [7]. They also showed no analgesia in the tail flick assay. Therefore, we assessed the analgesic effects of the novel compounds using the formalin injection nociception assay and the writhing test. Consistent with their μOR agonist activity, PZM21 and the novel compounds displayed dose-dependent analgesia in these two assays. The most potent compound **7d** exhibited 93.9% and 92.7% analgesia, respectively, at the low dose of 2.5 mg/kg. These results were higher than those of PZM21 at doses of 40 mg/kg and 10 mg/kg in the formalin injection and writhing assays, respectively.

The structure–activity relationship studies showed that replacing the phenolic hydroxyl group of PZM21 with a larger naphthalene group could maintain the μOR agonist activity but decrease the selectivity of β-arrestin-2 recruitment. Additionally, the position of both the naphthalene and thiophene rings affected the potency and efficacy. The (*R*) stereoisomer of **7c** had a potency that was reduced by 1000-fold, while the most potent compound**7d** was not chiral, suggesting a specific stereochemical requirement for both potency and efficacy.

Our studies support minimal β-arrestin-2 signaling in vitro by PZM21; however, its analgesic effect still needs to be further confirmed. PZM21 did not show an analgesic effect in the mouse hotplate assay, a traditional pain model; therefore, the painkilling activity of PZM21 remains ambiguous. Although novel compound **7b** displayed a higher efficacy in vivo than PZM21, respiratory depression induced by this compound still needs to be further investigated.

## 3. Experimental Section

### 3.1. General Information

Reagents were obtained from Beijing Chemical Works (Beijing, China), Acros Organics (Geel, Belgium), and Alfa-Aesar (Ward Hill, MA, USA) and were used without further purification. Proton nuclear magnetic resonance (^1^H-NMR, 400 MHz) spectra were measured with a JNM-ECA-400spectrometer (JEOL Co. Ltd., Tokyo, Japan). Mass spectra were measured by using an API-150 mass spectrometer (ABI Inc., Foster City, CA, USA) with an 1100-HPLC electrospray ionization source (Agilent Technologies Inc., Palo Alto, CA, USA).

### 3.2. Chemistry

#### 3.2.1. Synthesis **2a** and **2b**

Thionyl chloride (11.05 g, 92.92 mmol) was slowly added to a suspension of **1a** or **1b** (10.00 g, 46.46 mmol) in methanol (100 mL) in an ice bath. The mixture was allowed to warm up to room temperature and stirred for 12 h. The solvent was removed under reduced pressure. The obtained yellow solid was dissolved in ammonia solution (120 mL) and methanol (30 mL), and stirred for 12 h. The mixture was concentrated under reduced pressure. The obtained yellow solid was purified by silica gel column chromatography using dichloromethane-methanol (12:1) as the eluent, yielding **2a** (8.83 g, 83.82%) or **2b** (8.10 g, 76.89%) as a white powdery solid.

#### 3.2.2. Synthesis of **3a** and **3b**

Aqueous formaldehyde (22.00 mL, 294.00 mmol, 37%) was added to a suspension of **2a** or **2b** (5.30 g, 24.75 mmol) in methanol (20 mL), followed by the addition of 10% palladium carbon (2.00 g). The hydrogenation reaction was carried out for 5 h at a pressure of 0.4 MPa and room temperature. After the reaction was completed, palladium carbon was removed by suction filtration, and the filtrate was concentrated under reduced pressure. The crude residue was purified by silica gel column chromatography using dichloromethane-methanol (1:20) as the eluent, yielding **3a** (4.30 g, 71.78%) or **3b** (4.53 g, 75.62%) as a white solid.

#### 3.2.3. Synthesis of **4a** and **4b**

A 1M solution of borane-tetrahydrofurane complex (107.00 mL, 107.00 mmol) was slowly added to solution of **4a** or **4b** (4.30 g, 17.76 mmol) in anhydrous THF (50 mL). The mixture was refluxed for 8 h under a nitrogen atmosphere. The reaction was quenched with anhydrous methanol (50 mL, dropwise). The solvent was removed under reduced pressure. The obtained crude residue was purified by silica gel column chromatography using dichloromethane-methanol (50:1–20:1) as the eluent. The obtained oily residue was resuspended in methanol (20 mL) with the addition of excess HCl/diethyl ether and concentrated under reduced pressure, yielding **4a** (3.10 g, 58.16%) or **4b** (3.33 g, 62.48%) as a white solid.

#### 3.2.4. Synthesis of **6a**–**c**

Compounds **5a**–**c** (6.09 g, 34.40 mmol) and triethylamine (9.60 mL, 68.80 mmol) in anhydrous THF (150 mL) were mixed under a nitrogen atmosphere in an ice bath. A solution of 4-nitrophenyl chloroformate (6.93 g, 34.40 mmol) in anhydrous THF (60 mL) was added dropwise. The reaction mixture was allowed to warm up to room temperature and stirred for 2 h. The slurry was diluted with dichloromethane (100 mL) and filtered. The filtrate was washed and concentrated under reduced pressure. The crude residue was purified by silica gel column chromatography using petroleumether-dichloromethane (4:1–1:1) as the eluent, yielding **6a**–**c** as a white foam (6.04–6.44 g, 57.36–61.16%).

#### 3.2.5. Synthesis of **9**

Ethanolamine (17.00 mL, 280.00 mmol) was added dropwise to formic acid (15.00 mL, 400.00 mmol) in an ice bath. Nitroethane (26.00 mL, 360.00 mmol) and **8** (3.9 mL, 45 mmol) were added to the mixture. The reaction mixture was stirred for 7 h. The resulting solution was poured into cold water (500 mL), and the slurry was filtered. The precipitated product was recrystallized from ethanol/water (4:1 *v*/*v*, 50 mL), yielding **9** as a yellow crystalline solid (9.80 g, 64.47%).

#### 3.2.6. Synthesis of **10**

Compound **9** (9.00 g, 53.25 mmol) in anhydrous THF (120 mL) was added dropwise to a slurry of LiAlH_4_ (10.02 g, 266.25 mmol) in anhydrous THF (250 mL) in an ice bath. The mixture was stirred for 12 h under a nitrogen atmosphere. The reaction was quenched by the dropwise addition of water (10 mL) and 15% NaOH (10 mL). After filtration, the filter cake was washed with ethyl acetate. The residue was dissolved in diethyl ether (100 mL), cooled to 0 °C, and the product was precipitated by excess HCl/diethyl ether and filtered off. The precipitate was recrystallized from acetonitrile (200 mL), yielding **10** as a gray crystalline solid. 49 g, 79.01%).

#### 3.2.7. Synthesis of **5a** and **5b**

Racemic **10** (3.15 g, 17.70 mmol) was dissolved in water (100 mL), and the solution was basified by aqueous ammonia and extracted with dichloromethane (3 × 80 mL). The liquid was added to a hot solution of di-*p*-anisoyl-d-tartaric acid (7.50 g, 17.70 mmol) in acetonitrile (60 mL). The slurry was diluted with water (30 mL). The obtained crystalline precipitate was dissolved in 1N NaOH and extracted with dichloromethane. The organic layers were dried over anhydrous Na_2_SO_4_. The liquid residue was dissolved in diethyl ether (70 mL), cooled to 0 °C, and the product was precipitated by excess HCl/diethyl ether, yielding a white solid (2.13 g, 67.62%, ee = 34.0%). The above step was repeated three times, yielding **5a** as a white solid (0.8 g, 26.99%, ee = 98.4%).

Compound **5b** was obtained by the same procedures as those for **5a** except that di-*p*-anisoyl-l-tartaric acid was used as a chiral resolving agent, yielding **5b** as a white solid (0.7 g, 25.23%, ee = 98.0%).

#### 3.2.8. Synthesis of **7a**–**g**

To a suspension of **4a** or **4b** (0.60 g, 1.99 mmol) in acetonitrile (15 mL), triethylamine (0.90 mL, 5.97 mmol) was added. Next, a solution of **6a**–**c** (0.67 g, 2.19 mmol) in acetonitrile (15 mL) was added. The mixture was stirred for 2 h, filtered, and concentrated under reduced pressure. The residue was dissolved in ethyl acetate (20 mL) and filtered. The crude residue was purified by silica gel column chromatography using dichloromethane-methanol (100:1–30:1) as the eluent, yielding the desired product **7a**–**g** as a yellow oil (0.47–0.52 g, 59.49–65.82%).

1-((*S*)-2-(Dimethylamino)-3-(naphthalen-1-yl)propyl)-3-((*S*)-1-(thiophen-3-yl)propan-2-yl)urea (**7a**): ^1^H-NMR δ_H_ (400MHz, DMSO-d_6_, ppm): 0.91–0.93 (d, *J* = 6.44Hz, 3H, CH-CH_3_), 2.38 (s, 6H, N-CH_3_), 2.50–2.54 (m, 2H, Ar-CH_2_thiophene), 2.61–2.87 (m, 2H, Ar-CH_2_), 2.90–3.05 (m, 2H, CH_2_-NH), 3.34–3.49 (m, 1H, CH-N), 3.63–3.74 (m, 1H, NH-CH), 5.65–5.66 (d, *J* = 4.76Hz, 1H, NH-CO), 6.01–6.03 (d, *J* = 7.84Hz, 1H, NH-CO), 6.88–6.90 (dd, *J* = 1.12, 5.04Hz, 1H, H_Ar_), 7.07–7.08 (d, *J* = 1.96Hz, 1H, H_Ar_), 7.36–7.46 (m, 3H, H_Ar_), 7.50–7.61 (m, 2H, H_Ar_), 7.78–7.80 (d, *J* = 8.12Hz, 1H, H_Ar_), 7.92–7.94 (m, 1H, H_Ar_), 8.06–8.08 (d, *J* = 8.16Hz, 1H, H_Ar_). MS-HR (ESI) m/z: 396.2104 [M + H].

1-((*S*)-2-(Dimethylamino)-3-(naphthalen-2-yl)propyl)-3-((*S*)-1-(thiophen-3-yl)propan-2-yl)urea (**7b**): ^1^H-NMR δ_H_ (400MHz, DMSO-d_6_, ppm): 0.91–0.92 (d, *J* = 6.44Hz, 3H, CH-CH_3_), 2.30 (s, 6H, N-CH_3_), 2.54–2.67 (m, 2H, Ar-CH_2_thiophene), 2.75–2.89 (m, 2H, Ar-CH_2_), 2.98–3.10 (m, 2H, CH_2_-NH), 3.38–3.41 (m, 1H, CH-N), 3.67–3.73 (m, 1H, NH-CH), 5.63–5.65 (d, *J* = 5.04Hz, 1H, NH-CO), 6.00–6.02 (d, J = 7.84Hz, 1H, NH-CO), 6.90–6.91 (dd, *J* = 1.12, 4.76Hz, 1H, H_Ar_), 7.09 (d, *J* = 1.68Hz, 1H, H_Ar_), 7.36–7.40 (m, 2H, H_Ar_), 7.50–7.61 (m, 2H, H_Ar_), 7.70 (s, 1H, H_Ar_), 7.83–7.88 (m, 3H, H_Ar_). MS-HR (ESI) *m*/*z*: 396.2104 [M + H].

1-((*S*)-2-(Dimethylamino)-3-(naphthalen-2-yl)propyl)-3-((*R*)-1-(thiophen-3-yl)propan-2-yl)urea (**7c**): ^1^H-NMR δ_H_ (400MHz, DMSO-d_6_, ppm): 0.90–0.91 (d, *J* = 6.44Hz, 3H, CH-CH_3_), 2.30 (s, 6H, N-CH_3_), 2.46–2.69 (m, 2H, Ar-CH_2_thiophene), 2.74–2.92 (m, 2H, Ar-CH_2_), 2.98–3.13 (m, 2H, CH_2_-NH), 3.37–3.47 (m, 1H, CH-N), 3.68–3.76 (m, 1H, NH-CH), 5.65–5.66 (d, *J* = 4.76Hz, 1H, NH-CO), 6.02–6.04 (d, *J* = 7.88Hz, 1H, NH-CO), 6.93–6.95 (dd, *J* = 1.12, 4.76Hz, 1H, H_Ar_), 7.12 (d, *J* = 1.96Hz, 1H, H_Ar_), 7.36–7.43 (m, 2H, H_Ar_), 7.45–7.51 (m, 2H, H_Ar_), 7.70 (s, 1H, H_Ar_), 7.83–7.88 (m, 3H, H_Ar_). MS-HR (ESI) *m*/*z*: 396.2105 [M + H].

(*S*)-1-(2-(Dimethylamino)-3-(naphthalen-1-yl)propyl)-3-(2-(thiophen-3-yl)ethyl)urea (**7d**): ^1^H-NMR δ_H_ (400MHz, DMSO-d_6_, ppm): 2.38 (s, 6H, N-CH_3_), 2.58–2.62 (t, *J* = 7.14Hz, 2H, Ar-CH_2_thiophene), 2.78–2.83 (m, 2H, Ar-CH_2_), 2.94–3.05 (m, 2H, CH_2_-NH), 3.10–3.15 (q, 2H, CH_2_-NH), 3.30–3.40 (m, 1H, CH-N), 5.75 (s, 1H, NH-CO br), 6.09–6.12 (t, *J* = 5.20Hz, 1H, NH-CO), 6.94–6.95 (d, *J* = 4.8Hz, 1H, H_Ar_), 7.13 (s, 1H, H_Ar_), 7.37–7.46 (m, 3H, H_Ar_), 7.50–7.58 (m, 2H, H_Ar_), 7.78–7.80 (d, *J* = 8.12Hz, 1H, H_Ar_), 7.92–7.94 (d, *J* = 7.28Hz, 1H, H_Ar_), 8.06–8.08 (d, *J* = 8.12Hz, 1H, H_Ar_). MS-HR (ESI) *m*/*z*: 382.1948 [M + H].

(*S*)-1-(2-(Dimethylamino)-3-(naphthalen-2-yl)propyl)-3-(2-(thiophen-3-yl)ethyl)urea (**7e**): ^1^H-NMR δ_H_ (400MHz, DMSO-d_6_, ppm): 2.30 (s, 6H, N-CH_3_), 2.60–2.67 (t, *J* = 7.14Hz, 2H, Ar-CH_2_thiophene), 2.77–2.91 (m, 2H, Ar-CH_2_), 2.98–3.17 (m, 4H, CH_2_-NH), 3.30–3.40 (m, 1H, CH-N), 5.73–5.75 (d, *J* = 4.76Hz, 1H, NH-CO), 6.10–6.13 (t, *J* = 5.46Hz, 1H, NH-CO), 6.95–6.96 (d, *J* = 5.04Hz, 1H, H_Ar_), 7.14 (s, 1H, H_Ar_), 7.36–7.50 (m, 4H, H_Ar_), 7.70 (s, 1H, H_Ar_), 7.83–7.88 (m, 3H, H_Ar_). MS-HR (ESI) *m*/*z*: 382.1949 [M + H].

(*S*)-1-(2-(Dimethylamino)-3-(naphthalen-1-yl)propyl)-3-(2-(thiophen-2-yl)ethyl)urea (**7f**): ^1^H-NMR δ_H_ (400MHz, DMSO-d_6_, ppm): 2.38 (s, 6H, N-CH_3_), 2.78–2.87 (m, 4H, Ar-CH_2_), 2.91–3.05 (m, 2H, CH_2_-NH), 3.12–3.17 (q, 2H, CH_2_-NH), 3.32–3.36 (m, 1H, CH-N), 5.77–5.79 (m, 1H, NH-CO), 6.19–6.22 (t, *J* = 5.74Hz, 1H, NH-CO), 6.81–6.82 (q, 1H, H_Ar_), 6.90–6.93 (q, 1H, H_Ar_), 7.29–7.30 (dd, *J* = 1.12, 5.04Hz, 1H, H_Ar_), 7.37–7.39 (q, 1H, H_Ar_), 7.42–7.46 (t, *J* = 7.42Hz, 1H, H_Ar_), 7.50–7.58 (m, 2H, H_Ar_), 7.78–7.80 (d, *J* = 8.12Hz, 1H,H_Ar_), 7.92–7.94 (q, 1H,H_Ar_), 8.06–8.08 (d, *J* = 8.44Hz, 1H, H_Ar_). MS-HR (ESI) *m*/*z*: 382.1948 [M + H].

(*S*)-1-(2-(Dimethylamino)-3-(naphthalen-2-yl)propyl)-3-(2-(thiophen-2-yl)ethyl)urea (**7g**): ^1^H-NMR δ_H_ (400MHz, DMSO-d_6_, ppm): 2.30 (s, 6H, N-CH_3_), 2.74–2.92 (m, 4H, Ar-CH_2_), 2.97–3.10 (m, 2H, CH_2_-NH), 3.13–3.20 (m, 2H, CH_2_-NH), 3.35–3.47 (m, 1H, CH-N), 5.77–5.78 (d, *J* = 4.48Hz, 1H, NH-CO), 6.19–6.22 (t, *J* = 5.74Hz, 1H, NH-CO), 6.82–6.83 (q, 1H, H_Ar_), 6.91–6.94 (dd, *J* = 3.36, 5.04Hz, 1H, H_Ar_), 7.30–7.31 (dd, *J* = 1.12, 5.04Hz, 1H, H_Ar_), 7.36–7.39 (q, 1H, H_Ar_), 7.43–7.51 (m, 2H, H_Ar_), 7.70 (s, 1H, H_Ar_), 7.81–7.88 (m, 3H, H_Ar_). MS-HR (ESI) *m*/*z*: 382.1948 [M + H].

### 3.3. μOR G_i/o_-Mediated cAMP Inhibition

Human embryonic kidney (HEK) 293 cells in the logarithmic growth phase were washed with phosphate-buffered saline and dissociated with trypsin. Then, the cells were centrifuged, resuspended in serum-free culture media (containing 0.1% bovine serum albumin and 0.5 mM 3-isobutyl-1-methylxanthine), plated at a density of 2000 cells per 5-μLaliquotin 384-well cell culture plates, and incubated at 37 °C with 5% CO_2_. Drug dilutions (the final drug concentrations were 100 μM, 10 μM, 1 μM, 100 nM, 10 nM, 1 nM, 100 pM, and 0 μM) were prepared in saline or DMSO in triplicate. A 5 μL aliquot of each drug solution was added to each well, and the plates were incubated for 30 min in the dark at room temperature. Then, to increase the level of intracellular cAMP, 5 μL of forskolin (final concentration of 10 μM) was added to each well, and the plate was incubated for 30 min in the dark at room temperature. At the end of the reaction, the cAMP assay substrate was added to each well, and the cells were again incubated in the dark at room temperature for 60 min. The luminescence intensity was quantified using an EnVision 2104 microplate reader (PerkinElmer Inc., Waltham, CA, USA). Data were normalized to [d-Ala^2^, *N*-Me-Phe^4^, Gly^5^-ol]-enkephalin (DAMGO)-induced cAMP inhibition and analyzed with GraphPad Prism 6.0 software (Graphpad Software Inc., San Diego, CA, USA).

### 3.4. β-Arrestin Recruitment Assay

β-Arrestin recruitment was measured by a NanoBit assay, which is a double subunit system based on NanoLuc luciferase that can be used to detect intracellular protein interactions. The plasmids expressing MOR or ARRB1/2 and fused with the subunit LgBiT (17.6 kDa) or SmBiT(11 amino acids), respectively, were provided by the National Center for Drug Screening (Shanghai, China). HEK293 cells were cotransfected with the plasmids stated above by electric shock for at least 24 h. Next, the transfected cells were plated into 96-well, white, opaque bottom, cell culture plates in culture media at a density of 2 × 10^5^ cells in 50 μL of medium and incubated at 37 °C with 5% CO_2_ overnight. Then, 40 μL of phenol-red-free medium and 10 Μl of substrate were added to each well, and the plate was incubated for 10 min in the dark. Finally, 50 μL of drug solution was added to three wells, and the plate was incubated for 10 min in the dark. The plates were read using an EnVision 2104 micro plate reader (PerkinElmer Inc., Waltham, CA, USA). The data were normalized to DAMGO-induced stimulation and analyzed using nonlinear regression in GraphPad Prism 6.0 software (Graphpad Software Inc., San Diego, CA, USA).

### 3.5. Formalin Injection Assay

Analgesia to formalin injection was carried out as described previously [15]. Male adult ICR mice, weighing 25 ± 2 g, from Vital River Laboratory Animal Technology Co. Ltd. (Beijing, China) were used. The mice had free access to water and food, and were kept at 21 ± 2 °C with a relative humidity of 60 ± 10% and illumination for 12 h/day (8 a.m. to 8 p.m.). The mice were acclimated for 2 days and randomly divided into nine groups. Each group of mice was first habituated for 20 min in our homemade polyvinyl chloride cage without bedding, food, or water. Then, vehicle (0.1 mL of saline/10 g of body weight, *n* = 8), PZM21 (40 mg/kg, *n* = 8), or **7a**–**g** (40 mg/kg, *n* = 8) was injected subcutaneously. After 1 h, 20 μL of 2.7% formalin in saline was injected under the skin of the dorsal surface of the right hind paw, and the mice were returned to their cage. Nociception was estimated by measuring the cumulative amount of time spent by the animals licking the formalin-injected paw during the late phase (20–30 min). The analgesic effect of the compounds was presented as the percentage of the inhibition ratio, according to the following formula:

(1) $$\%Analgesia=\frac{\mathrm{Average}\;\mathrm{Time}\left(\mathrm{Vehicle}\right)-\mathrm{Average}\;\mathrm{Time}\left(\mathrm{Drug}\right)}{\mathrm{Average}\;\mathrm{Time}\left(\mathrm{Vehicle}\right)}\times 100\%$$

### 3.6. Analgesic Activitiesin the Writhing Test

The writhing test methods described by Souza et al. [16] were followed, with a few modifications. Male adult ICR mice, weighing 25 ± 2 g, from Vital River Laboratory Animal Technology Co. Ltd. (Beijing, China) were used. The mice had free access to water and food, and were kept at 21 ± 2 °C with a relative humidity of 60 ± 10% and illumination for 12 h/day (8 a.m. to 8 p.m.). The mice were acclimated for 2 days and randomly divided into nine groups. Then vehicle (0.1 mL of saline/10 g of body weight, *n* = 8), PZM21 (10 mg/kg, *n* = 8), **7a**–**c** (10 mg/kg, *n* = 8), **7e**–**g** (10 mg/kg, *n* = 8), or **7d** (2.5 mg/kg, *n* = 8) was injected subcutaneously. After 0.5 h, 1% acetic acid solution (10 mL/kg) was injected intraperitoneally, and the mice were returned to their cage. The number of writhes exhibited by each mouse was counted for 20 min. The analgesic effect of the compounds was presented as the percentage of the inhibition ratio, according to the following formula:

(2) $$\%Analgesia=\frac{\mathrm{Average}\;\mathrm{Number}\left(\mathrm{Vehicle}\right)-\mathrm{Average}\;\mathrm{Number}\left(\mathrm{Drug}\right)}{\mathrm{Average}\;\mathrm{Number}\left(\mathrm{Vehicle}\right)}\times 100\%$$

## 4. Conclusions

In conclusion, novel PZM21 analogues were synthesized and evaluated for their specificity and efficacy as μOR-biased agonists. Novel compound **7a** and PZM21 were confirmed to show undetectable β-arrestin-2 recruitment. However, several novel compounds exhibited significant analgesic activity in the formalin injection assay and in the writhing test. Compound **7d** was more potent than PZM21 and exhibited the most potent analgesic activity of all of the novel compounds in the formalin injection and writhing assays, respectively. Therefore, compound **7d** should be used as lead to develop new biased μOR agonists for treating pain.

## Figures and Tables

**Table 1:**
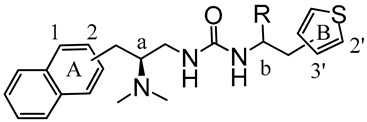
EC_50_ and Max Response of the tested compounds in the G_i_ activation assay.

Compound	Substituted Position of the -CH_2_- in the A Ring	Substituted Position of the -CH_2_- in the B Ring	R and Configuration of Chiral Carbon Atom b	EC_50_(nM)	%Max Response (100 μM)
**7a**	1	3′	CH_3_ (*S*)	455.6	60
**7b**	2	3′	CH_3_ (*S*)	91.14	80
**7c**	2	3′	CH_3_ (*R*)	>100,000	-
**7d**	1	3′	H	203.4	50
**7e**	2	3′	H	82.43	60
**7f**	1	2′	H	1448	40
**7g**	2	2′	H	242.8	60
PZM21	-	-	-	52.41	60
DAMGO	-	-	-	8.17	100

**Table 2:**
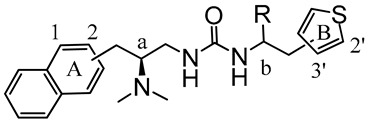
EC_50_ and Max Response of the tested compounds in the β-arrestin recruitment assay.

Compound	Substituted Position of the -CH_2_- in the A Ring	Substituted Position of the -CH_2_- in the B Ring	R and Configuration of Chiral Carbon Atom b	EC_50_ (μM)	%Max Response (100 μM)
**7a**	1	3′	CH_3_ (*S*)	N.A.^a^	0
**7b**	2	3′	CH_3_ (*S*)	0.7239	50
**7d**	1	3′	H	1.621	70
**7e**	2	3′	H	1.128	25
**7g**	2	2′	H	2.162	12
PZM21	-	-	-	N.A.^a^	0
DAMGO	-	-	-	1.237	100

^a^ No measurable activity.

**Table 3:**
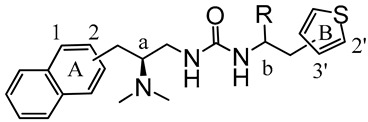
In vivo activities of the synthesized compounds in the formalin injection assay.

Compound	Substituted Position of the -CH_2_- in the A Ring	Substituted Position of the -CH_2_- in the B Ring	R and Configuration of Chiral Carbon Atom b	%Analgesia (20 mg/kg)	%Analgesia (40 mg/kg)
**7a**	1	3′	CH_3_ (*S*)	53.7 ± 17.8 **	76.9 ± 19.1 **
**7b**	2	3′	CH_3_ (*S*)	82.4 ± 15.8 **	96.7 ± 3.4 **
**7c**	2	3′	CH_3_ (*R*)	79.6 ± 17.2 **	53.5 ± 19.9 **
**7d**	1	3′	H	100 **	100 **
**7e**	2	3′	H	84.1 ± 12.5 **	92.3 ± 12.9 **
**7f**	1	2′	H	69.6 ± 21.7 **	86.7 ± 9.0 **
**7g**	2	2′	H	89.8 ± 10.6 **	91.7 ± 7.4 **
PZM21	-	-	-	77.9 ± 14.6 **	92.8 ± 11.2 **

Values are the mean ± standard deviation (*n* = 8). ** *p* < 0.001 versus control.

**Table 4:**
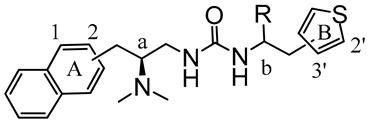
In vivo activities of the synthesized compounds in the writhing test.

Compound	Substituted Position of the -CH_2_- in the A Ring	Substituted Position of the -CH_2_- in the B Ring	R and Configuration of Chiral Carbon Atom b	Start Time of Writhing (s)	%Analgesia
**7a**	1	3′	CH_3_ (*S*)	307	60.6 ± 10.5 **
**7b**	2	3′	CH_3_ (*S*)	525	65.1 ± 13.4 **
**7c**	2	3′	CH_3_ (*R*)	261	27.1 ± 10.6 **
**7d**	1	3′	H	891	92.7 ± 9.7 **
**7e**	2	3′	H	320	42.4 ± 13.4 **
**7f**	1	2′	H	317	44.4 ± 8.6 **
**7g**	2	2′	H	334	50.3 ± 10.5 **
PZM21	-	-	-	254	45.7 ± 15.0 **

Values are the mean ± standard deviation (*n* = 8). ** *p* < 0.001 versus control. Compound **7d** was administered at a dose of 2.5 mg/kg; the others were administered at a dose of 10 mg/kg.
